# The VWFA Is the Home of Orthographic Learning When Houses Are Used as Letters

**DOI:** 10.1523/ENEURO.0425-17.2019

**Published:** 2019-02-15

**Authors:** Lea Martin, Corrine Durisko, Michelle W. Moore, Marc N. Coutanche, Deborah Chen, Julie A. Fiez

**Affiliations:** 1 Department of Psychology; 2 Center for the Neural Basis of Cognition; 3 Learning Research and Development Center, University of Pittsburgh, Pittsburgh, PA 15260; 4 Department of Communication Sciences and Disorders, West Virginia University, Morgantown, WV 26506; 5 Robert Wood Johnson Medical School, Rutgers University, New Brunswick, NJ 08901

**Keywords:** fMRI, language learning, left fusiform gyrus, linguistic bridge account, reading development

## Abstract

Learning to read specializes a portion of the left mid-fusiform cortex for printed word recognition, the putative visual word form area (VWFA). This study examined whether a VWFA specialized for English is sufficiently malleable to support learning a perceptually atypical second writing system. The study utilized an artificial orthography, HouseFont, in which house images represent English phonemes. House images elicit category-biased activation in a spatially distinct brain region, the so-called parahippocampal place area (PPA). Using house images as letters made it possible to test whether the capacity for learning a second writing system involves neural territory that supports reading in the first writing system, or neural territory tuned for the visual features of the new orthography. Twelve human adults completed two weeks of training to establish basic HouseFont reading proficiency and underwent functional neuroimaging pre and post-training. Analysis of three functionally defined regions of interest (ROIs), the VWFA, and left and right PPA, found significant pre-training versus post-training increases in response to HouseFont words only in the VWFA. Analysis of the relationship between the behavioral and neural data found that activation changes from pre-training to post-training within the VWFA predicted HouseFont reading speed. These results demonstrate that learning a new orthography utilizes neural territory previously specialized by the acquisition of a native writing system. Further, they suggest VWFA engagement is driven by orthographic functionality and not the visual characteristics of graphemes, which informs the broader debate about the nature of category-specialized areas in visual association cortex.

## Significance Statement

Fluent reading recruits a portion of the brain known as the visual word form area (VWFA), but it is less well understood how malleable the VWFA remains after acquiring literacy in a native language. There is also debate about the type of visual information the VWFA can process as orthographically meaningful. We tested whether native English-speaking adults could learn a second, visually atypical writing system for English and used neuroimaging data to assess the location of any learning effects. Participants acquired basic reading ability and learning effects were found in the neural territory that underlies English reading. This suggests that the VWFA remains plastic after initial literacy and is not restricted by the visual features of a writing system.

## Introduction

Acquiring a second language in adulthood is challenging, in part because neural resources become specialized for native language processing (Tan et al., 2003; Hull and Vaid, 2007). This specialization can make it difficult to use the same neural tissue to support fluency in a second language (Mårtensson et al., 2012; Klein et al., 2014). In this article, we examined a related question: to what degree can adults acquire a second writing system for their native language? To address this question, we taught adult native English speakers a perceptually atypical artificial orthography for English. We used behavioral and functional magnetic resonance imaging (fMRI) methods to ascertain if their newly learned reading skill involved a region already specialized for reading English, the putative visual word form area (VWFA).

The VWFA is a region in the left fusiform gyrus that preferentially responds to orthographic visual stimuli (Cohen et al., 2002; McCandliss et al., 2003; Cohen and Dehaene, 2004; Szwed et al., 2011; Glezer et al., 2015; but for alternative accounts of the VWFA, see Price and Devlin, 2003; Vogel et al., 2014). This response specialization emerges with the acquisition of literacy (Saygin et al., 2016), even when native language literacy is acquired in adulthood (Dehaene et al., 2010), suggesting an absence of a “critical” period of plasticity (Bornstein, 1989).

Less is known about the degree to which the VWFA remains plastic once it has become specialized to support a native writing system, and to what extent its recruitment depends on the perceptual characteristics of a writing system. The widespread acquisition of second language literacy suggests the VWFA can support skilled reading for multiple orthographies (Tschirner, 2016). However, this apparent ease may be misleading due to the high degree of visual similarity between naturally occurring orthographies (Hirshorn and Fiez, 2014). This visual similarity may reflect the cultural evolution of writing systems to use forms that are optimized for the representational capacities of the VWFA (Dehaene, 2009), in which case, the VWFA may be poorly equipped to respond to a perceptually atypical orthography. Further, the high degree of visual similarity between natural writing systems may allow any literacy-driven specialization of the VWFA to readily transfer to another orthography, thereby overestimating the plasticity of the VWFA for orthographies that are perceptually distant from the native orthography.

A strong test of the VWFA’s plasticity therefore requires acquisition of a perceptually atypical orthography by an individual whose VWFA has already been specialized by a native orthography. The need to disentangle factors that are intertwined in naturally occurring orthographies motivates the use of an artificial orthography in the present study. We build on a previously reported study that used face images as “letters” to represent English phonemes (Moore et al., 2014b). In this previous study, orthographic learning effects were observed in the left mid-fusiform cortex, but there was ambiguity whether these effects localized to the VWFA or to tissue specialized for face processing, the left fusiform face area (FFA). Thus, it remains unclear whether orthographic learning effects localize to tissue that is specialized for processing the visual characteristics of the grapheme forms (e.g., words printed with face letters to the FFA) or whether visual stimulus with orthographic functionality may induce plasticity within the VWFA, even when it has already been specialized for a perceptually typical native orthography.

To address this question, we trained English speakers to read an artificial orthography in which images of houses represent English phonemes (HouseFont). We chose houses because they are preferentially processed in a region known as the parahippocampal place area (PPA), which is spatially distant from the VWFA. The PPA’s distinctiveness allows us to identify the neural tissue dedicated to processing the graphemes of our new orthography. We employed a localizer scan to functionally identify the PPA and VWFA, and pre-training and post-training scans to isolate neural changes associated with HouseFont learning. This allowed for a clear test of whether a VWFA tuned to a native orthography (English) has the flexibility to respond to a second orthography (HouseFont), even when this second orthography uses graphemes that are highly distinctive from those used in the Roman alphabet. If the perceptual characteristics of grapheme forms drive the locus of orthographic learning, significant learning effects should be observed in the PPA. Alternatively, if the functional use of visual forms as orthographic symbols drives the locus of orthographic learning, and the neural tissue that supports this learning remains malleable, significant learning effects should be observed in the VWFA.

## Materials and Methods

### Participants

Fourteen University of Pittsburgh undergraduate students were originally enrolled in the study. This sample size was selected based on research showing that imaging research can achieve power of roughly 80% using a threshold of 0.05 and 12 subjects (Desmond and Glover, 2002), and results for our prior study (Moore et al., 2014b) in which significant differences in the VWFA territory were observed for between-group comparisons (*N* = 11 and *N* = 12) of the response to a trained versus untrained orthography. One participant dropped out on the second day of training and one dropped out after having completed everything except the post-training imaging session. Data from the final sample of 12 individuals (eight female, four male) are reported (M age = 19.17 years, SD = 1.19). All participants were recruited from a database of individuals interested in participating in research studies. All study participants were right-handed, native English speakers, and had no history of second language fluency, hearing or vision issues, learning or reading problems, drug or alcohol abuse, mental illness, neurologic issues, or contraindications for fMRI. All participants provided informed consent and were compensated for their time. All procedures were approved by the institutional review board of the University of Pittsburgh.

### Study overview

**Figure 1.**
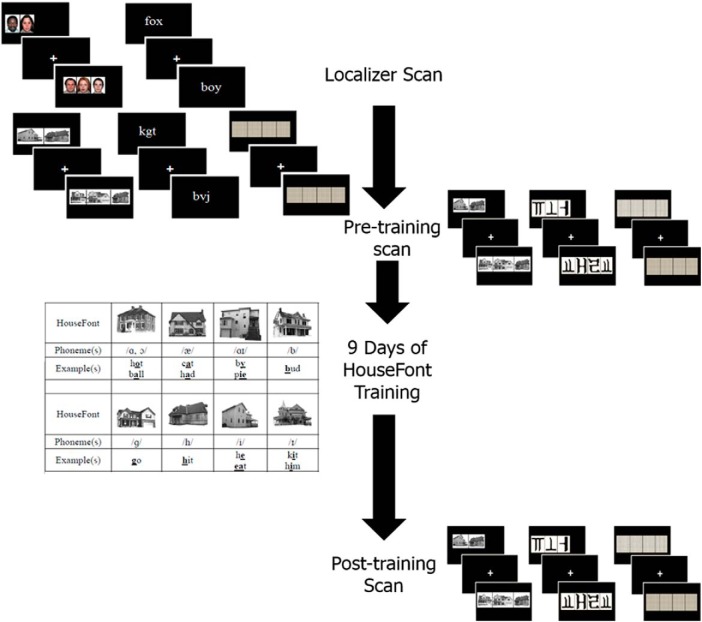
Participants completed a localizer scan, a pre-training scan, HouseFont training, and a post-training scan. The images alongside each point on the timeline are examples of the stimuli used for the neuroimaging sessions.

The study involved a two-week training protocol to learn HouseFont. Training occurred after two pre-training fMRI sessions and before a post-training fMRI session. The first of the pre-training fMRI sessions was designed to localize three regions of interest (ROIs): the VWFA and the left and right PPA. The purpose of the second pre-training fMRI session was to measure the response to words printed in HouseFont before training. The final fMRI session measured the response to HouseFont after training. Behavioral measures of post-training reading skill were also acquired as part of this final session. Participants were debriefed and paid following the post-training scan. Figure 1 provides an overview of the study timeline and the design of specific tasks. Table 1 summarizes the HouseFont training protocol. Further details are provided below.

**Table 1.** HouseFont training protocol

Week	Session	Tasks
Baseline		Localizer fMRI
		Pre-training fMRI
Week 1	Session 1	Phoneme training Phoneme test
	Session 2	Phoneme training review Word level training Word test (1)
	Session 3–5	Word level training Word test (2–4)
Week 2	Session 6–9	Story level training Word test (5–8)
	Session 10	Reading test (GORT-4) Post-training fMRI

### Pre-training fMRI sessions

#### Localizer session

Participants started the study by completing a localizer fMRI session and a battery of standardized reading tests. The localizer session was conducted using a Siemens Medical Systems 3T Magnetom TIM Trio scanner with a 32-channel radio frequency coil. High-resolution structural scans were collected using an axial MPRAGE with 192 slices and 1-mm isotropic voxels. Functional data were collected across 29 interleaved slices in the same plane as the structural data (TR = 1500 ms, TE = 25 ms, FOV = 200 mm, FA = 70**°**).

During functional data acquisition, participants completed a 1-back task with five categories of visual stimuli: (1) houses, (2) faces, (3) words, (4) letter-strings, and (5) patterns (Fig. 1). Following similar localizer protocols used in prior studies (Fox et al., 2009; Rossion et al., 2012), stimuli were drawn from sets of 40 exemplars for each of the non-orthographic (houses, faces, and patterns) categories, and sets of 157 exemplars for the orthographic (word and letter-string) categories. The scan consisted of four functional runs each lasting 6 min. Every run had a total of 15 blocks (three of each category, randomly ordered). Blocks consisted of 15 trials, with the stimulus for each trial presented for 200 ms followed by an 800-ms fixation cross. Participants were asked to press a key when they detected a stimulus that repeated the one shown previously (i.e., 1-back). A 1-back target was presented for 12.5% of each block. A 9-s baseline condition followed each block. During this baseline, participants attended to a fixation cross at the center of the screen. During each run, the sets of house, face, and pattern stimuli were distributed pseudorandomly within each of the three blocks for each condition. With the exception of 1-back trials, the word and letter-string stimuli did not repeat. None of the house images used in the localizer task were used as stimuli in the subsequent parts of the study.

#### Pre-training session

The pre-training scan was completed within a week of the localizer session. For logistic reasons, the scanner, a 3T Siemens Allegra equipped with a standard radio frequency coil, differed from that used for the localizer session. High-resolution structural scans were collected using a sagittal MPRAGE with 192 slices and 1-mm isotropic voxels. Functional data were collected across 38 interleaved slices (3.125 × 3.125 × 3.2 mm voxels) parallel to the anterior-posterior commissure (TR = 2000 ms, TE = 25 ms, FOV = 200 mm, FA = 70**°**).

During the pre-training scan participants passively viewed 140 words printed in HouseFont and an untrained artificial orthography, KoreanFont. KoreanFont is an artificial alphabetic orthography that borrows graphemes from Hangul, the Korean writing system, and assigns them to English phonemes. They also saw 16 pattern displays that were repeated over 140 trials. Word and pattern stimuli were matched for length. Participants completed two runs, which consisted of seven blocks of each stimuli type for a total of 21 blocks. Each block contained 10 trials of the same stimulus type. For each trial, participants saw one HouseFont or KoreanFont word or pattern set for 1500 ms, followed by 500 ms of a centrally located fixation cross (Fig. 1). They were instructed to attend to the stimuli, but were not asked to perform an overt task. The same set of HouseFont words were presented during the pre-training and post-training sessions; individuals were not exposed to this set of HouseFont words at any other time.

### HouseFont training

HouseFont consists of 35 grapheme-to-phoneme mappings, where each grapheme is a particular house image that is used to represent a single phoneme or (in a few cases) two very similar sounds (e.g., /ɑ/ in *hot* and /ɔ/ in *ball*). All of the house images used for HouseFont were 300 × 300 pixels, normalized, and lightened to a light gray. Participants were trained to read HouseFont across nine sessions, which were broken into three phases: house-phoneme mapping (session 1), word-level training (sessions 2–5), and story-level training (session 6–9). Each training session lasted from 1 to 2 h. These training phases are summarized.

#### Session 1: house-phoneme mapping

Participants began their training by learning to map each HouseFont grapheme with a corresponding phoneme using a self-paced computer program. The 35 house graphemes were visually presented in random order, and participants pressed a spacebar to hear the corresponding sound after each grapheme was displayed. Participants completed five cycles of the phoneme training, followed by a test of their ability to produce the phoneme associated with each grapheme. Participants who achieved <90% accuracy repeated the training. All participants passed in three or fewer attempts.

#### Sessions 2–5: word-level training

After a brief refresher on the house-phoneme mapping, participants learned how to read aloud short words printed in HouseFont. Each session of the word-level training involved reading 400 one- to two-syllable words, which were two to five phonemes in length. The same set of 400 words was used in sessions 2–5, with the word order randomized across sessions. For each trial, participants were encouraged to attempt to read the word when it appeared; they had the option to hear any individual phoneme or the entire word if necessary. At the end of each session, a computer-based, single-word-reading test was administered. Each word test consisted of three conditions presented in a block design, with the order of blocks randomized across test sessions: old HouseFont words (words included in word-level training), new HouseFont words, and pronounceable HouseFont non-words. There were 20 trials per condition. A trial consisted of a one-syllable word that was three to four phonemes in length. The pronunciation accuracy was scored for each item, and reading latency was measured from the time a word first appeared on the screen to when the participant pressed the space bar to advance to the next word.

#### Sessions 6–9: story-level training

**Figure 2.**
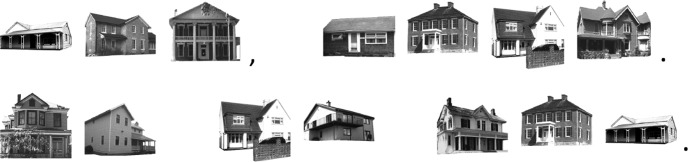
An example of part of a story printed in HouseFont. It reads, “Look, Father. See the ball.”

In the final training stage, participants advanced to reading aloud short stories printed in HouseFont (Fig. 2). For each session, participants read 10 early reader stories of similar difficulty from the “Now I’m Reading!” series (Gaydos, 2003). The story level increased in difficulty with each successive session. Performance on story reading was measured by words read per minute. At the end of each session, participants completed a single-word-reading test identical in design and scoring to those used during word-level training.

### Post-training behavioral and fMRI session

During the final session (session 10), participants completed behavioral testing to assess their final HouseFont reading skill and an fMRI session to measure learning-related changes in the neural response to HouseFont. For the behavioral testing, participants’ reading speed and accuracy were assessed using six passages (Form A Stories 1–6) from the gray oral reading test-4 (GORT-4; Wiederholt and Bryant, 2001) that were transcribed into HouseFont. Number of words read per minute and number of errors made per word were calculated as an index of reading speed and accuracy respectively. The number of errors made per word was determined by dividing the number of errors (e.g., omissions, phoneme substitutions, whole word or part word repetitions, etc.) made by the number of words in each passage. The post-training scan was completed during session 10 immediately after administration of the behavioral tests, using the same scanner and fMRI protocol as in the pre-training scanning session.

### fMRI data analysis

#### fMRI data preprocessing

Preprocessing of the fMRI data were completed using the Analysis of Functional NeuroImages (AFNI) software package (Cox, 1996). The first two brain volumes from the localizer runs and the first brain volume from the pre-training and post-training runs were removed to allow for stabilization of the signal. The functional images were slice time corrected (3dTshift), and all data were motion corrected (3dvolreg). The data were smoothed using a Gaussian filter set to a smoothing kernel of 5.5-mm full width at half maximum. Next, the functional images were registered to the skull stripped high-resolution structural images. Images were then transformed into standard Talairach space using a non-linear warping procedure in AFNI to allow for group analysis (Talairach and Tournoux, 1988). Functional images were scaled to a mean global intensity.

#### ROIs identification

The central question of this study is whether HouseFont learning is supported by neural tissue specialized by the acquisition of a native (English) orthography (i.e., territory at or near the VWFA) or tissue that shows selectivity for the perceptual characteristics of the non-native HouseFont orthography (i.e., the territory at or near the PPA). To address this question, the data from the localizer session were used to functionally localize a priori ROIs in the left fusiform and bilateral parahippocampal cortices.

Multivariate pattern analysis (MVPA) was used to identify each of the three ROIs within MATLAB using the Princeton Multi-Voxel Pattern Analysis toolbox (Detre et al., 2006). For this analysis, the functional data preprocessing was the same as described above, with one exception: as is common in MVPA, the data were not spatially smoothed (Mur et al., 2009). MVPA has been found to be more sensitive to fine grain differences between stimuli (for review, see Coutanche, 2013). This increased sensitivity allowed us to successfully localize the left fusiform ROI using the hallmark contrast used in early work characterizing the VWFA: words and letter-strings (Petersen et al., 1990; Cohen et al., 2002; Dehaene et al., 2002). To localize the PPA ROIs, a house and word contrast was used.

For each run, we *z* scored the pre-processed activity values for each voxel, accounting for the hemodynamic delay by shifting the condition time course by two TRs. A Gaussian naive Bayes (GNB) classifier was trained and tested on the activity patterns for the contrasts of interest (words versus letter-strings and houses versus words) using a leave-one-run-out cross-validation procedure, where each iteration was trained on data from all-but-one run (e.g., three runs), and tested on data from the held-out run. Classification performance from the iterations was averaged to give a single accuracy value. The resulting accuracy for the contrasts (where chance is 50%) was then allocated to the central voxel of a three-voxel radius searchlight sphere, which was moved serially across the brain.

We identified the voxel with peak decoding accuracy for the words versus letter-strings contrast within AFNI’s anatomic mask of the left fusiform cortex and for the houses versus words contrast within anatomic masks of the left and right parahippocampal cortex for each subject. To generate the group level ROIs for the VWFA and PPAs, we created a 6-mm radius sphere centered on the location of average peak accuracy across all subjects for the respective contrast in each anatomic mask (Table 2).

**Table 2.** Functionally defined ROIs that were applied to the pre-training and post-training data

Localizer ROI	Cluster size (voxels)	Center of mass coordinates (*x*,*y*,*z*)
Left parahippocampal gyrus (L PPA)	33	–28, –43, –7
Right parahippocampal gyrus (R PPA)	33	26, –43, –4
Left fusiform gyrus (VWFA)	33	–34, –55, –13

Coordinates are in Talairach space.

### Analysis of behavioral and neural learning effects

#### Analysis of behavioral learning effects

To test whether participants showed improvements in HouseFont reading during training, reading accuracy and reading speed were assessed for each of the word tests. A one-way repeated measures ANOVA was performed on the average reading latency scores for correct responses across the eight-word tests to determine whether reading speed changed over the course of training.

#### Analysis of neural training effects

To test whether participants showed neural changes associated with training (i.e., changes in the neural responses to HouseFont words), the pre-training and post-training data were modeled using AFNI’s 3dDeconvolve to estimate the BOLD response (average beta-weight value) for HouseFont and KoreanFont. The motion estimates from preprocessing were included as regressors of no interest. Then, we compared the resulting t-values for HouseFont and KoreanFont across the pre-training and post-training sessions, using both an ROI-based and a whole-brain (voxel-wise) group analysis.

For the ROI analysis, the VWFA and PPA ROIs identified from the localizer (Table 2) were applied to the pre- and post-training session data. Using AFNI 3dROIstats, the averaged beta weight value for the voxels within each ROI was obtained for each participant’s response to HouseFont and KoreanFont before and after HouseFont training. These values were exported to IBM Statistical Package for the Social Sciences (SPSS) version 25. To determine whether there were training and ROI based differences in HouseFont activation, a 2 × 2 × 3 repeated measures ANOVA was performed with orthography (HouseFont, KoreanFont), session (pre-training, post-training), and region (VWFA, left PPA, and right PPA) specified as within-subject variables. It was expected that there would be a significant three-way interaction, which would suggest there was a differential change in HouseFont activation between ROIs that resulted from HouseFont reading training. A significance threshold of *p* < 0.05 was used, with correction for all violations of normalcy in the data.

As a complementary analysis approach, a whole-brain voxel-wise analysis was used to identify pre-training versus post-training changes in the response to HouseFont without a priori constraints. The computed *t* values for the HouseFont versus KoreanFont contrast for each participant were contrasted across the pre-training versus post-training sessions for each voxel using AFNI 3dClustSim, with a significance threshold of *p* = 0.005 (corrected *p* = 0.05) and a cluster size threshold of 60 contiguous voxels.

#### Relationship between behavioral and neural measures

To examine the relationship between behavioral and neural measures of learning, each participant’s reading speed score from the final word test was standardized and combined with the standardized reading speed score from the GORT-4. This composite reading speed score was examined using a regression analysis, to determine whether the pre-training versus post-training change in the estimated BOLD responses within the VWFA ROI accounted for HouseFont reading speed variability.

Because the sample size of the current study is small, we performed a similar analysis that combined data from the participants in the current study (*N* = 12) with data from two participant groups reported by Moore et al. (2014b): one group that learned an artificial orthography with face images as letters (FaceFont; *N* = 12) and one group that learned an artificial orthography with borrowed Korean graphs mapped to English phonemes (KoreanFont; *N* = 11). For each participant from the Moore et al., study, the final reading speed was calculated in the same way as it was for HouseFont, by averaging the *z* score of the GORT reading speed and the inverse *z* score of the final word test reading speed. The imaging data from the Moore et al. study were acquired using the same design and scanner as in the current study, with the exception that only a post-training session was acquired, and instead of viewing HouseFont and KoreanFont words, participants viewed FaceFont and KoreanFont words. Because the data from the Moore et al., study were previously analyzed using a different software package, they were reprocessed using the same methods as in the current study.

Next, we used an ROI analysis to extract the average estimated BOLD response within the VWFA territory for each participant across our three groups (HouseFont-trained, FaceFont-trained, KoreanFont-trained). To avoid biasing the results by using the VWFA ROI identified using data from only the HouseFont participants, we drew on the literature to define an unbiased ROI for this across-group analysis. Specifically, we used a coordinate from a recent study by Lerma-Usabiaga et al. (2018), where real words and consonant strings were contrasted to localize a specific VWFA subregion in the middle occipitotemporal sulcus (mOTS) that exhibits lexical-level orthographic selectivity, and which can be distinguished from a more posterior VWFA subregion that is more generally responsive to visual word forms (pOTS). The average peak coordinate reported by Lerma-Usabiaga et al. (2018) for their mOTS subregion were rounded to the closest whole numbers, transformed into Talairach space, and used as a center of a 6-mm sphere (–42, –57, –4). Using AFNI 3dROIstats, the averaged beta weight value for the voxels within this mOTS ROI was obtained for each participant’s response to their trained orthography during the post-training scan. These values were entered into a regression analysis, along with the orthography learned by the participant, to predict participants’ reading speed following training.

## Results

### Behavioral measures of HouseFont learning

Average accuracy for trained participants across all of the word tests performed during training was 90%. This is not surprising, because HouseFont is a transparent orthography and so once the grapheme-phoneme mappings have been mastered, they can in theory be used to decode English words and pronounceable nonwords with perfect accuracy. For this reason, the focus of the behavioral training analyses was reading latency. To test whether participants showed improvements in HouseFont reading over the course of their training, a one-way repeated measures ANOVA was performed on the average reading latency score for correct responses on the eight-word tests. Two individuals were missing a single word test and were excluded from the analysis. The Greenhouse-Geisser correction was applied because Mauchly’s test of sphericity was not met, *p* = 0.01. There was a significant effect of test session *F*_(2.28,20.48)_ = 10.47, *p* = 0.001, which reflects a decrease in reading latencies over the course of HouseFont training. From the first word test (session 2) to the final word test (session 9), the average reading latency dropped from 6288 ms (SD = 1963 ms) to 4670 ms (SD = 1126 ms). This 25% reduction in reading latency indicates that participants became more skilled at reading HouseFont across the two weeks of training.

**Figure 3.**
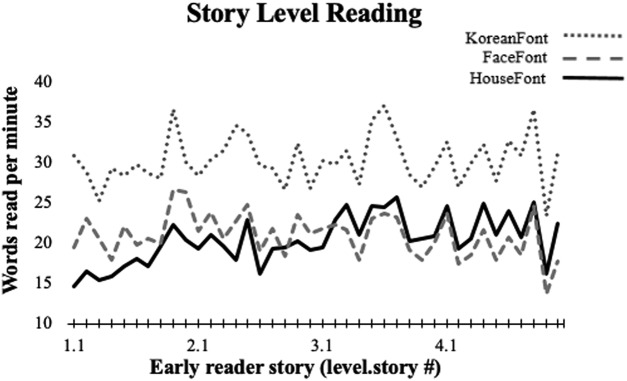
Stories increased in difficulty over the 4 d of story-level reading, but participants maintained a similar rate of words read per minute. The performance of HouseFont participants on the early reader training stories was consistent with performances seen for other artificial orthographies, KoreanFont, and FaceFont. KoreanFont and FaceFont data adapted with permission from Moore et al. (2014b).

Improvements in HouseFont reading were also evident in the context of story reading. Participants maintained a relatively steady rate of reading across story level training (sessions 6–9), although the stories became increasingly more difficult across sessions (Fig. 3). By the end of story-level training (session 9), participants were reading an average of 21.85 words per minute (SD = 2.88). Participants also read six passages of a standardized reading assessment, the GORT, to assess final reading accuracy and speed. On this measure participants attained a mean fluency of 21.15 (SD = 5.13) words per minute, with a mean error rate of 2% (SD = 0.02) per word. These proficiency results are similar to those observed for first grade children learning English (Hasbrouck and Tindal, 2006).

### Neural measures of HouseFont learning

#### ROI analysis

A 2 × 2 × 3 repeated measures ANOVA was used to examine the effect of orthography (HouseFont, KoreanFont), session (pre-training, post-training), and region (VWFA, left PPA, and right PPA) on neural activity. This analysis revealed a main effect of orthography, *F*_(1,11)_ = 97.07, *p* < 0.001, $\eta_{p}^{2}$ = 0.90, and region, *F*_(1.37,22)_ = 7.97, *p* = 0.008, $\eta_{p}^{2}$ = 0.42, with no effect of session, *F*_(1,11)_ = 0.11, *p* = 0.749, $\eta_{p}^{2}$ = 0.01. There was a significant interaction between orthography and region, *F*_(1.79,22)_ = 10.41, *p* = 0.001, $\eta_{p}^{2}$ = 0.49, and trend level interactions for orthography and session, *F*_(1,11)_ = 4.32, *p* = 0.062, $\eta_{p}^{2}$ = 0.28, and training and region, *F*_(1.49,22)_ = 3.20, *p* = 0.079, $\eta_{p}^{2}$ = 0.23. Most importantly, the predicted three-way interaction was also significant, *F*_(1.44,22)_ = 6.25, *p* = 0.016, $\eta_{p}^{2}$ = 0.36.

**Figure 4.**
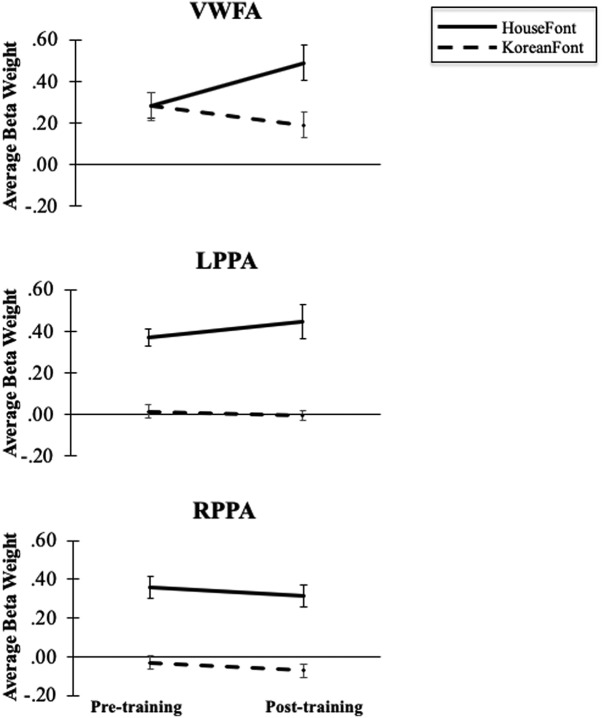
The VWFA showed no main effect for session or orthography, but there was a significant interaction of session and orthography. The left and right PPA showed the expected significant main effect of orthography, no main effect of training, and no significicant interaction between session and orthography. Error bars indicate SE.

To examine the three-way interaction and address our a priori hypothesis that HouseFont-elicited activity in the VWFA would change after training, we ran a separate 2 × 2 repeated measures ANOVA [orthography (HouseFont, KoreanFont), session [pre-training, post-training] for each region. Within the VWFA there was a main effect of orthography, *F*_(1,11)_ = 15.23, *p* = 0.002, $\eta_{p}^{2}$ = 0.58 and no effect of session, *F*_(1,11)_ = 0.86, *p* = 0.374, $\eta_{p}^{2}$ = 0.07 (Fig. 4). Critically, however, there was a significant interaction between orthography and session, *F*_(1,11)_ = 9.79, *p* = 0.010, $\eta_{p}^{2}$ = 0.47, in the VWFA. *Post hoc* comparisons of the interaction revealed that the response to KoreanFont decreased across sessions, *p* = 0.100, while HouseFont evoked greater activation in the post-training session compared to pre-training session, *p* = 0.059. These are the expected results if the HouseFont training tuned the VWFA to treat strings of HouseFont images as orthographic information.

In the left PPA, there was an effect of orthography, *F*_(1,11)_ = 55.43, *p* < 0.001, $\eta_{p}^{2}$ = 0.83, no effect of session, *F*_(1,11)_ = 0.47, *p* = 0.507, $\eta_{p}^{2}$ = 0.04, and no significant interaction between orthography and session, *F*_(1,11)_ = 1.91, *p* = 0.194, $\eta_{p}^{2}$ = 0.15. Similarly, in the right PPA, there was an effect of orthography, *F*_(1,11)_ = 62.12, *p* < 0.001, $\eta_{p}^{2}$ = 0.85, no effect of session, *F*_(1,11)_ = 1.31, *p* = 0.276, $\eta_{p}^{2}$ = 0.11, and no interaction between orthography and session, *F*_(1,11)_ = 0.00, *p* = 0.993, $\eta_{p}^{2}$ = 0.00. The expected main effects of orthography and the lack of other effects show that the PPA bilaterally responded more to HouseFont than KoreanFont and that HouseFont training did not alter this difference.

#### Whole-brain voxel-wise analysis

**Figure 5.**
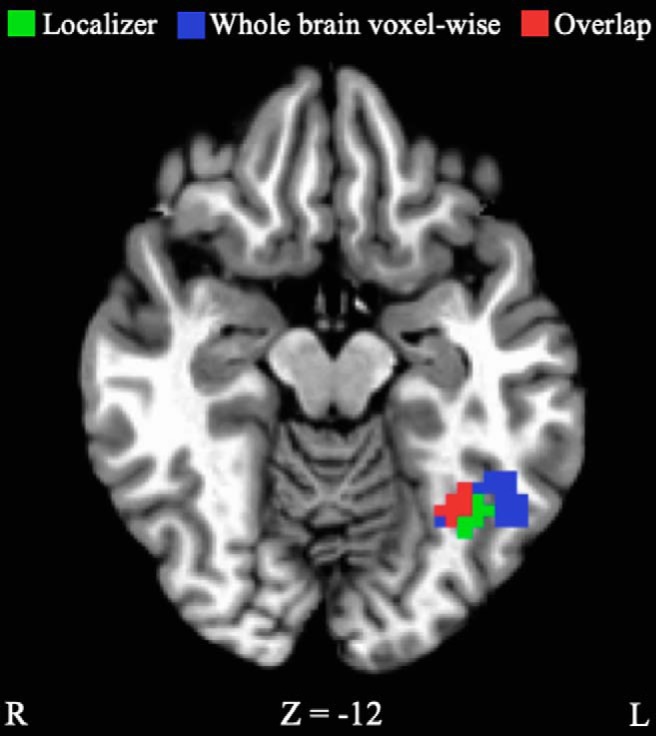
VWFA ROI (green) identified by the localizer scan (–34, –55, –13), and the learning effect cluster (blue) identified from the whole-brain voxel-wise analysis of activation for HouseFont versus KoreanFont from pre-training to post-training (–40, –49, –10). Red represents the overlap. Coordinates are in Talairach space.

To investigate whether HouseFont training altered the response to HouseFont strings in areas outside of the a priori ROIs, a whole-brain voxel-wise analysis was conducted with the pre-training and post-training fMRI data. HouseFont activation was compared to KoreanFont activation in both the pre-training and post-training scans separately. Then, the difference in pre-training was compared to the difference in post-training. This comparison yielded 10 significant training effect clusters, nine of which were negative, indicating more activation in post-training. The one positive cluster, which was located in the left middle temporal gyrus (BA19), indicates more activation during pre-training (Table 3). Several of the clusters are in regions known to be involved in reading (Bolger et al., 2005), including the left inferior frontal gyrus, the left superior parietal lobe, and the left fusiform gyrus. Portions of the left fusiform gyrus training effect cluster overlapped with the VWFA ROI (Fig. 5), which is not surprising given the significant interaction effect found in the VWFA ROI. No training effect clusters were identified within the left or right parahippocampal gyrus.

**Table 3.** Clusters identified by the whole-brain voxel-wise analysis [trained orthography (HouseFont) vs untrained orthography (KoreanFont), pre-training to post-training]

Cluster location	Cluster size (voxels)	Peak coordinates (*x*,*y*,*z*)
Left superior parietal lobe (BA7)	418	–28, –64, 44
Left precentral/inferior frontal gyrus (BA6/BA8)	322	–49, 2, 14
Right posterior cerebellum	233	17, –64, –22
Left thalamus/left caudate nucleus	197	–7, –13, 14
Right caudate	95	17, 14, 14
Left medial frontal gyrus (BA6)	95	–1, 14, 44
Left middle frontal gyrus (BA46)	81	–43, 29, 20
Left middle temporal gyrus (BA19)*	72	–49, –61, 17
Left fusiform gyrus (BA37)	68	–40, –49, –10
Left insula (BA13)	65	–31, 17, 11

All clusters were identified with a corrected *p* = 0.05. Coordinates are in Talairach space.

BA, Brodmann area; * indicates the cluster that displayed more activation during pre-training.

### Relationship between behavioral and neural measures of HouseFont learning

**Figure 6.**
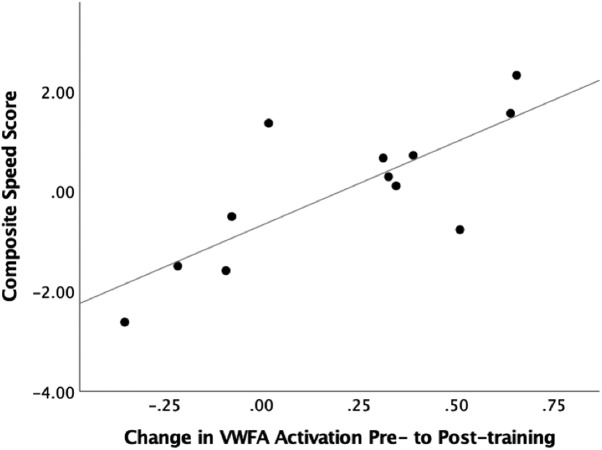
Scatter plot of the variance explained by the pre-training to post-training change of the VWFA for reading speed. The VWFA change showed a significant positive relationship with reading speed. Reading speed scores were zero centered.

To probe the relationship between neural and behavioral measures of HouseFont learning effects, we performed a regression to test the contribution of training related activation change in the VWFA to HouseFont reading speed. A HouseFont reading speed score was calculated by averaging the *z* score of the number of words read per minute on the GORT and the inverse *z* score (*z* score multiplied by –1) of the response time per word on the final word test. The change in activation from pre-training to post-training in the VWFA did significantly predict reading speed *b* = 3.34, *t*_(10)_ = 3.90, *p* = 0.003, and it explained a significant proportion of variance in reading speed scores, *R* ^2^ = 0.60, *F*_(1,10)_ = 15.24, *p* = 0.003 (Fig. 6). Based on these results, we conclude that the VWFA is critical for rapid HouseFont reading.

**Figure 7.**
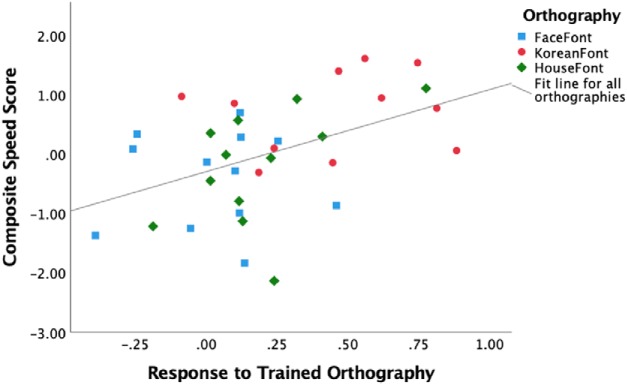
Scatter plot of the variance in reading speed explained by the response to trained orthography within the VWFA ROI. The response to the trained orthography showed a significant positive relationship with reading speed. Reading speed scores were zero centered across all three orthographies.

We obtained convergent results using data from the HouseFont-trained participants in the current study, and the FaceFont-trained and KoreanFont-trained participants previously reported by Moore et al. (2014b). While the three orthographies differ in the graphs they use and in their average reading speed (Fig. 3), we expected that behavioral measures of reading speed would be significantly predicted by the VWFA activation in response to the trained orthography. We assessed this using a specific VWFA subregion reported in the literature (mOTS; Lerma-Usabiaga et al., 2018) as an ROI (to avoid biasing our ROI localization to the HouseFont group). The post-training response to the trained orthography within the mOTS ROI significantly predicted reading speed *b* = 1.38, *t*_(32)_ = 2.82, *p* = 0.008. On the other hand, which orthography a participant learned (FaceFont, KoreanFont, or HouseFont) did not significantly predict reading speed *b* = –0.00, *t*_(32)_ = –0.01, *p* = 0.992. These results align with previous reports of FaceFont and KoreanFont learning effects (Moore et al., 2014b) and the findings from HouseFont. Moreover, the significant relationship between the neural and behavioral measures of learning suggest that despite the visual differences in the graphs used, reading speed variation across all three artificial orthographies can be predicted by learning effects seen within the VWFA territory (Fig. 7).

## Discussion

This study tested whether acquisition of a perceptually atypical second writing system recruits the same neural tissue already tuned by native-English reading, or whether instead the locus of orthographic learning tracks with the perceptual characteristics of the grapheme forms. More specifically, we were interested in the presence or absence of artificial orthography (HouseFont) learning effects within three functionally defined areas: an orthographic area (VWFA) within the left mid-fusiform gyrus (Cohen and Dehaene, 2004), and bilateral place areas (left PPA, right PPA) within the parahippocampal gyri (Epstein and Ward, 2010). We hypothesized that orthographic learning effects would be observed in either the VWFA or the PPA, but not in both regions. Significant learning effects were found only within the VWFA, and individual differences in the magnitude of pre-training versus post-training changes in VWFA activation correlated with differences in HouseFont reading speed. We conclude the VWFA was recruited to support HouseFont literacy acquisition in our adult participants.

The results from this study converge with Moore et al. (2014b), who also observed training-related increases in the VWFA territory when participants learned one of two artificial alphabets for English: FaceFont, in which face images were used as letters, and KoreanFont, in which letters were borrowed from the Korean alphabet and mapped to English phonemes. Taken together, the results from the current study and Moore et al. (2014b) point toward three principles of VWFA function: (1) learning a new alphabetic orthography uses VWFA tissue already specialized by acquisition of English literacy, (2) orthographies with a wide range of visual forms can induce neural plasticity in the VWFA, (3) the laterality of the VWFA is influenced by the mapping principles of an orthography.

### New orthographic learning uses the same tissue as English

The HouseFont training effects demonstrate that the VWFA in native English speakers was modified by HouseFont learning. Similarly, Moore et al. (2014b) found a left-lateralized training effect for FaceFont in the vicinity of the VWFA. However, they could not conclusively assign FaceFont learning to the same territory that supports English reading for two reasons. First, a putative left homolog of the right-lateralized face processing area (Kanwisher et al., 1997) falls in close proximity to the VWFA (Nestor et al., 2013). Consequently, the locus of observed FaceFont learning effects could arguably reflect the use of neural tissue specialized for face or orthographic processing. Second, Moore et al. (2014b) did not localize the response to printed English in their participants, so they were unable to directly compare the functional response to English and FaceFont. The present study circumvented these problems by using house graphs associated with category-specific activation in tissue that is spatially distant from the VWFA and by functionally localizing the VWFA before HouseFont training.

While we attribute the change in HouseFont activation within the VWFA to orthographic learning, alternative accounts warrant consideration. It is possible that repetitive exposure to a small set of visual images could be sufficient to increase the VWFA response to the frequently experienced images. We cannot completely discount this possibility because none of our studies have involved a control group with similar exposure to the image sets in a non-literacy context. However, we favor the idea that the activation changes in the VWFA are related to literacy acquisition. This is because the regions in which activation increased were selective, the learning effects in the fusiform gyrus correlate with reading (Fig. 6; Moore et al., 2014b), and the connectivity of the VWFA is suited for visual-phonological mapping (Alvarez and Fiez, 2018).

It is also important to remember that imaging is a correlational, rather than a causal, method. It is possible that part or all of the increased VWFA activation following training could be from accessing the English orthographic representations of the HouseFont words. If this were the case, it could mean the VWFA is not necessary for accurate HouseFont reading, but rather is activated as a by-product of accurately decoding the HouseFont word. We took extra care to ensure that HouseFont graphemes were never equated with an English grapheme and no English appeared during the training phase. Additionally, prior work with artificial orthographies found that a patient with acquired alexia was unable to learn a small set of face-phoneme pairings but was able to learn face-syllable pairings (Moore et al., 2014a). This finding suggests that the VWFA territory is critical rather collateral to learning an artificial alphabetic orthography.

### Visual and brain constraints on orthographic learning

Our findings also demonstrate that there is considerable flexibility in the type of visual forms that can serve as letters of an alphabet. This is not a trivial point, as this observed flexibility is counter to some theories of how the brain and reading shape one another. Most notably, Dehaene (2009; p 184) conjectured that orthographies have culturally evolved to be visually similar to each other because they are forced to conform to the abilities of the available neural tissue. As part of this argument, Dehaene specifically suggested that both face and house images are avoided almost entirely by writing systems because the VWFA, which supports skilled reading, is not the preferred processing area for this kind of visual information (Dehaene, 2009). The findings of this study, and those of Moore et al. (2014b), challenge this idea, because they show that participants can readily obtain basic reading proficiency for an orthography with perceptually atypical forms (house or face images).

One potentially important caveat is that individuals tend to read FaceFont and HouseFont more slowly than an artificial orthography made of more typical graphs (KoreanFont; Fig. 3). This could reflect intrinsic limitations, such as those posited by Dehaene (2009). Alternatively, it could reflect differences in the visual complexity and discriminability of faces and houses, as compared to the simpler and higher-contrast letter forms in KoreanFont, or that tissue tuned for printed English might better transfer this tuning to a visually similar orthography (e.g., KoreanFont) as compared to a visually dissimilar (e.g., FaceFont, HouseFont) orthography. Transfer effects also might occur for other characteristics of an orthography, such as its grouping of graph elements (such as the dots in Arabic words; Abadzi, 2012). This transfer effect hypothesis could be tested by comparing the learning of artificial orthographies in which graphemes are borrowed from natural orthographies varying in perceptual distance from a reader’s native orthography. For example, we might predict native English speakers would read an artificial orthography with Korean graphemes more quickly than one with Arabic graphemes because Korean letters are more visually similar to English letters.

Despite baseline differences in reading speed, similar rates of learning are found across HouseFont, FaceFont, and KoreanFont (Fig. 3) and there is no evidence of a learning plateau across six weeks of training (Martin et al., 2018). Taken together, these results support Moore et al. (2014b)’s conclusion that tuning of the VWFA for English creates a “perceptual bottleneck” that slows the visual discrimination of a perceptually atypical second orthography, without preventing accurate reading and fluency gains with continued reading experience. In sum, the weight of evidence suggests that learnable orthographies are not constrained by the brain, but instead that experience with an orthography shapes the brain.

### Laterality effects in orthographic learning

Finally, our results demonstrate that alphabetic orthographic learning recruits left-lateralized brain regions, regardless of the perceptual characteristics of the orthography. In the whole-brain voxel-wise analysis, a strong pattern of left-lateralized regions showed HouseFont training effects (Table 3), and a similar set of regions showed training effects in FaceFont (unpublished findings). Most notably, both the current study and Moore et al. (2014b) found training effects in the left fusiform gyrus. The lack of a training effect in the right fusiform gyrus in Moore et al. (2014b) is particularly striking as face processing has been associated with right-lateralized visual processing (Kanwisher et al., 1997; Grill-Spector et al., 2004).

HouseFont, FaceFont, and KoreanFont differ visually, but share the same alphabetic mapping principle. To clarify whether the principle of left-lateralization holds true for non-alphabetic orthographies, we turn to Hirshorn et al. (2016)’s Faceabary training study in which face images represented English syllables. The study found Faceabary training effects in both the left and right mid-fusiform gyrus, with more bilateral patterns of activation correlated with higher Faceabary reading fluency. In contrast, Hirshorn et al. (2016) found a strong pattern of left-lateralization outside of the fusiform gyrus when comparing pre-training to post-training activation for Faceabary, which is consistent with results from both the current study and Moore et al. (2014b). This leads us to conclude that a key driver of left-lateralized fusiform gyrus recruitment is whether an orthography implements an alphabetic mapping principle, while a broader left-lateralized reading network is recruited irrespective of an orthography’s mapping principle.

## Conclusions

The current study found that adult acquisition of a perceptually atypical alphabetic orthography induced left-lateralized neural plasticity in the VWFA. We conclude that the VWFA remains highly malleable in adulthood. Further, our results, in combination with other work, indicate that the localization of orthographic learning to the VWFA is driven by orthographic functionality rather than the visual characteristics of a script, while the lateralization of the VWFA is influenced by the mapping principles of a script.

## Synthesis

Reviewing Editor: Philippe Tobler, University of Zurich Decisions are customarily a result of the Reviewing Editor and the peer reviewers coming together and discussing their recommendations until a consensus is reached. When revisions are invited, a fact-based synthesis statement explaining their decision and outlining what is needed to prepare a revision will be listed below. Note: If this manuscript was transferred from JNeurosci and a decision was made to accept the manuscript without peer review, a brief statement to this effect will instead be what is listed below.

Thank you for a responsive revision. A couple of minor points I noticed:

Figure 2 may be slightly confusing for non-specialists in that the same house symbol appears to be used for superficially different elements in the English language (e.g., the ‘re’ in ‘here’ and the ‘er’ in ‘father’). A clarifying explanation would be helpful.

Lines 304/305 include an instance of ‘a the’. Please pick one or the other.
